# Ethnic inequalities in involuntary admission under the Mental Health Act: an exploration of mediation effects of clinical care prior to the first admission

**DOI:** 10.1192/bjp.2022.141

**Published:** 2023-01

**Authors:** Daniela Fonseca Freitas, Susan Walker, Patrick Nyikavaranda, Johnny Downs, Rashmi Patel, Mizanur Khondoker, Kamaldeep Bhui, Richard D. Hayes

**Affiliations:** Institute of Psychiatry, Psychology and Neuroscience, King's College London, UK and Department of Psychiatry, University of Oxford, UK; Division of Psychiatry, University College London, UK and Great Ormond Street Institute of Child Health, University College London, UK; Department of Primary Care & Public Health, Brighton & Sussex Medical School, University of Sussex, UK; Institute of Psychiatry, Psychology and Neuroscience, King's College London, UK and South London and Maudsley NHS Foundation Trust, UK; Norwich Medical School, University of East Anglia, UK; Department of Psychiatry, University of Oxford, UK and Nuffield Department of Primary Care Health Sciences, University of Oxford, UK; Institute of Psychiatry, Psychology and Neuroscience, King's College London, UK

**Keywords:** ethnicity, health inequities detention, involuntary hospital admission, community mental healthcare

## Abstract

**Background:**

Studies show ethnic inequalities in rates of involuntary admission and types of clinical care (such as psychological therapies). However, few studies have investigated if there is a relationship between clinical care practices and ethnic inequalities in involuntary admission.

**Aims:**

This study investigated the impact of ethnicity and clinical care on involuntary admission and the potential mediation effects of prior clinical care.

**Method:**

In this retrospective cohort study, we used data from the electronic records of the South London and Maudsley NHS Foundation Trust and identified patients with a first hospital admission between January 2008 and May 2021. Logistic regression and mediation analyses were used to investigate the association between ethnicity and involuntary admission, and whether clinical care, in the 12 months preceding admission, mediates the association.

**Results:**

Compared with White British people, higher odds of involuntary admission were observed among 10 of 14 minority ethnic groups; with more than twice the odds observed among people of Asian Chinese, of Asian Bangladeshi and of any Black background. There were some ethnic differences in clinical care prior to admission, but these had a minimal impact on the inequalities in involuntary admission. More out-patient appointments and home treatment were associated with higher odds of involuntary admission, whereas psychological therapies and having a care plan were associated with reduced odds of involuntary admission.

**Conclusions:**

Ethnic inequalities in involuntary admission persist after accounting for potential mediating effects of several types and frequencies of clinical care. Promoting access to psychological therapies and ensuring that care plans are in place may reduce involuntary admissions.

## The Mental Health Act

Half of people involuntarily admitted for mental healthcare under the Mental Health Act 1983 (MHA) stated that this was the best care they could have had at the time, and 5% considered it a lifesaving experience.^1^ But about a third of people who were detained under the MHA considered this to be a negative, unhelpful, humiliating and traumatising experience.^1^ Detention increases perceptions of unfair treatment^2^ and can be considered a violation of human rights.^3^ An involuntary admission is related to more use of coercive control methods, such as seclusion,^4^ and coercive methods decrease patients’ satisfaction with care and can lead to negative perceptions of the therapeutic relationship.^5^ Thus, for some people, in the long run, detention under the MHA can lead to poorer healthcare outcomes and further involuntary admissions.^6,7^

## Ethnic inequalities in care pathways

People of minoritised ethnic groups, such as Black and Asian, are more likely to have a compulsory admission than White British people.^8^ Research has focused on the referral pathways into specialised mental healthcare and involuntary admission, with people from Black and Asian groups identified as having more adverse pathways into secondary care, more police involvement and less input from primary care.^9^ However, few studies have focused on care provided prior to admission. Lack of engagement with services is one reason commonly given to justify the ethnic inequalities, albeit unsupported by the available evidence.^8^

Studies also show minoritised ethnic groups living with psychosis are less likely to receive cognitive–behavioural therapy (CBT).^10,11^ Among those referred to talking therapies (via the Improving Access to Psychological Therapies (IAPT) programme), minoritised ethnic groups are less likely to receive an assessment.^12^ CBT does not seem to be associated with rates of involuntary admissions, according to a review of randomised controlled trials, although some of the trials lacked statistical power to investigate the association.^13^ Research using real-world data is needed.

## Clinical care and involuntary admission

There is some evidence that receiving care from a crisis team, or a home treatment team, is associated with reduced involuntary hospital admissions.^13,14^ Interventions that enhance shared decision-making, such as developing a crisis plan, reduced involuntary hospital admissions in randomised controlled trials.^15,16^ In one study, prior recent contact with community mental health services was associated with lower odds of involuntary admission among people with psychosis.^4^ Further research is needed to investigate if ethnic inequalities in involuntary admission may be partially explained by reduced access to these potentially protective therapeutic interventions.

The aim of this study was to investigate whether clinical care prior to hospital admission explains some of the ethnic differences in rates of involuntary admission. More precisely, we investigate if the association between ethnicity and involuntary admission is mediated by the type and frequency of clinical care in the 12 months preceding admission. Although the study is exploratory in nature, based on the literature about inequalities in access to timely and appropriate care,^8,12^ our hypotheses are that minoritised ethnic groups are less likely to receive care from community services and that this is related to higher involuntary admission rates.

## Method

### Setting and data sources

In this retrospective cohort study, we use data from the electronic health records (EHRs) of the South London and Maudsley NHS Foundation Trust (SLaM). SLaM provides secondary mental healthcare for 1.3 million people (2018 estimates) living in four South London boroughs. The Clinical Record Interactive Search (CRIS) system allows access to information from the records since mid-2006.^17^ In addition to accessing information on structured fields from the electronic records, natural language processing algorithms are used to retrieve information in the free-text fields.^17^

CRIS received approval for use as a de-identified data-set for secondary data analysis from the Oxford C Research Ethics Committee (18/SC/0372).^17^ Approval for this project was obtained from the service user-led CRIS oversight committee (19-066). Given the use of routinely collected data, active patient consent for publication is not required. Data from people who chose not to share their health data for research (national data opt-out) were excluded from the study.

### Sample inclusion and exclusion criteria

This study sample is a cohort of people admitted for:

(a) in-patient care for the first time between 1 January 2008 and 31 May 2021;

(b) aged 18 years and over at the time of admission;

(c) living in Greater London at the time of admission; and

(d) had a personal address or general practice address in the SLaM catchment area during the study's observation window (1 January 2007 to 31 May 2021).

We excluded people who were sectioned under the MHA Part III (concerning criminal proceedings) at any point of their admission as forensic sections have different pathways into them. We also excluded people with incomplete data on gender, age or ethnicity. Among people with a complete admission episode between 1 January 2008 and 31 May 2021, 4.6% had no information on ethnicity.

### Outcome

This study's outcome measure was involuntary admission to hospital, defined as admission under MHA sections 2, 3, 4 or 5(2) within 2 days of admission. The 2-day window was allowed to accommodate potential delays in updating the records.

### Predictors of interest

Ethnicity was coded based on the ethnic categories used by the National Health Service (NHS) classifications, and we only merged data (because of small sample sizes) of people identified as White – Gypsy/Irish Traveller, Other ethnic group – Arab and any Other ethnic group into the Other ethnicity group.

Based on the literature, as well as available information on the CRIS system, we considered several forms of clinical care in the 12 months prior to admission. This included: the total number of appointments with out-patients SLaM teams (categorised as 0, 1–5, 6–11 and ≥12 appointments because of skewed distribution and potential non-linearity); care received from a home treatment team, an early intervention for psychosis team and any psychological therapy provision. The latter comprised receiving care from the IAPT service (via linkage to the IAPT electronic patient database) or CBT in SLaM.^18^ As a result of a low frequency of appointments in these specialised services, these were coded as binary variables. Also, we analysed if patients had a record of a care plan.

### Potential confounders

Potential confounders included other sociodemographic information and psychiatric diagnoses. Sociodemographic confounders included gender, age on admission, migration, evidence of homelessness in the 12 months before admission and neighbourhood deprivation.

Migration was derived from information available from structured fields concerning the country of origin, first language, if the person needed an interpreter and applications for asylum or visa. Homelessness meant the person had no fixed abode at the time of admission or in the previous 12 months, or was reported with unstable housing or homelessness in a risk assessment (filled in between 12 months before admission and 28 days after admission). Neighbourhood deprivation was assessed using the Index of Multiple Deprivation of the English Indices of Deprivation 2010–2019, based on the patients’ address at the time of admission.

We included the psychiatric diagnoses (using the ICD-10) presented in the structured fields of the EHRs within the 28 days before and 28 after the admission date. These comprise the following groups of disorders: organic (F00–F09), substance use (F10–F19), schizophrenia spectrum (F20–F29), affective psychosis (F30.2, F31.2, F31.5, F32.3, F33.3), non-psychotic mood disorder (F30–F39, except the affective psychosis codes), stress-related disorders (F40–F48), behavioural syndromes related to physical factors (F50–F59), personality (F60–F69), mental disability (F70–79) disorders of psychological development (F80–F89) and behaviour/emotional disorders with onset in childhood (F90–F98).

### Statistical analysis

Logistic regression analyses were performed to investigate the association between ethnicity and clinical care, adjusting for confounders. As sensitivity analyses, we excluded people whose residency in the SLaM catchment area in the year prior to admission was uncertain and people who were admitted before 2010, due to potential inconsistency in the recording of service provision in the EHRs. Path analyses, using generalised structural equations modelling,^19^ investigated potential mediation effects of clinical care for the association between ethnicity and involuntary admission, while adjusting for confounders. See Supplementary Figure 1 (available at: http://dx.doi.org/10.1192/bjp.2022.141), for a diagram of the models tested. Indirect (or mediated) effects were only investigated among the ethnic groups where analyses showed a significant association between ethnicity and the measures of clinical care after adjusting for demographic and clinical factors. Also, only the clinical care factors significantly associated with involuntary admission were tested as potential mediators. Indirect effects were tested using bootstrap simulation based on 200 replications.

Statistical analyses were conducted using Stata 15. Because of the potential risk of bias in excluding people from the cohort without information on neighbourhood deprivation at the time of admission (12%) or migration (32%), missing data for these variables were coded as a category (undetermined).

## Results

### Participants

**Fig. 1:**
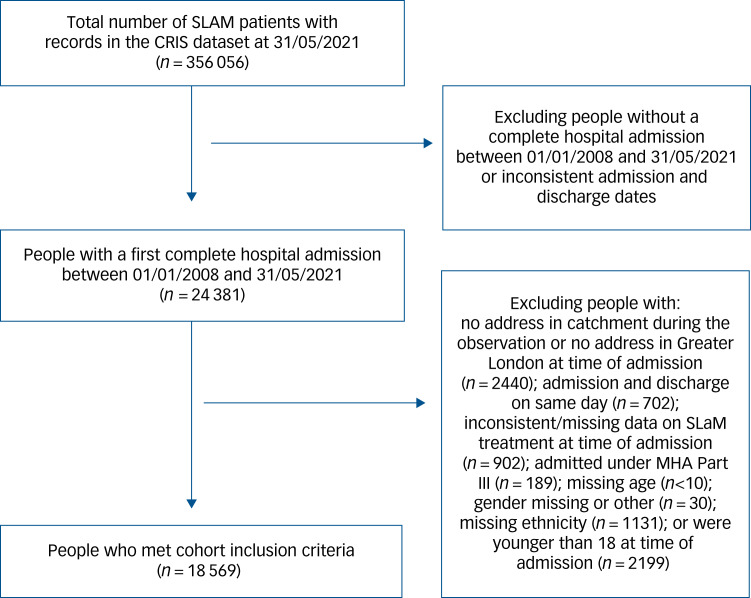
Cohort flow chart. MHA, Mental Health Act.

The sample comprised of 18 569 patients who met the inclusion criteria (see Fig. 1 for a flow diagram). The most prevalent ethnic groups were White British (44%), followed by Black African (11%), Black British (10%), Other White (10%), Black Caribbean (6%) and Asian British (4%) people. Patients’ median age at time of first admission was 39 years; 56% of the cohort were men.

The most prevalent diagnoses in the sample were schizophrenia spectrum disorder (20%), substance use related disorder (16%) and mood disorder (15%). In Table 1, a complete sample description is presented. Clinical and demographic characteristics stratified by ethnicity are presented in Supplementary Table 1.

**Table 1:** Demographic and clinical characteristics of the cohort of people admitted to hospital^a^

	Total	Admitted to hospital under the MHA
Total, *n* (%)	18 569 (100)	6437 (34.7)
Ethnicity, *n* (%)		
White British	8240 (44.4)	1873 (22.7)
White Irish	513 (2.76)	128 (25.0)
Other White background	1852 (10.0)	743 (40.1)
Black African	2031 (10.9)	1050 (51.7)
Black Caribbean	1145 (6.2)	576 (50.3)
Black British/Other Black background	1887 (10.2)	921 (48.8)
Asian Indian	287 (1.6)	104 (36.2)
Asian Pakistani	159 (0.9)	67 (42.1)
Asian Bangladeshi	90 (0.5)	42 (46.7)
Asian Chinese	130 (0.7)	68 (52.3)
Asian British/Other Asian background	661 (3.6)	263 (39.8)
White and Black African	83 (0.5)	26 (31.3)
White and Black Caribbean	227 (1.2)	68 (30.0)
Other Mixed race	171 (0.9)	56 (32.8)
Other ethnic background	1093 (5.9)	452 (41.4)
Gender, *n* (%)		
Men	10 343 (55.7)	3548 (34.3)
Women	8226 (44.3)	2889 (35.1)
Age, years: median (IQR)	39.1 (28.5–51.7)	38.2 (27.4–52.1)
18–24, *n* (%)	3006 (16.2)	1214 (40.4)
25–34, *n* (%)	4571 (24.6)	1603 (35.1)
35–49, *n* (%)	5835 (31.4)	1787 (30.6)
60–64, *n* (%)	2958 (15.9)	1026 (34.7)
65–99, *n* (%)	2199 (11.8)	807 (36.7)
Migrant (evidence of migration), *n* (%)		
No	7513 (40.5)	1898 (25.3)
Yes	5099 (27.5)	2161 (42.4)
Undetermined	5957 (32.1)	2378 (39.9)
Homeless 12 months prior admissions, *n* (%)		
No	15 058 (81.1)	5780 (38.4)
Yes	3511 (18.9)	657 (18.7)
Neighbourhood deprivation (IMD quintiles in sample), *n* (%)		
Least deprived quintile	2437 (13.1)	718 (29.5)
2nd least deprived	3367 (18.1)	1183 (35.1)
Middle deprivation	3566 (19.2)	1262 (35.4)
2nd most deprived	3607 (19.4)	1311 (36.4)
Most deprived quintile	3387 (18.2)	1216 (35.9)
Undetermined	2205 (11.9)	747 (33.9)
Diagnoses of psychiatric disorder, *n* (%)		
Organic disorder		
No	17 724 (95.5)	6128 (34.6)
Yes	845 (4.6)	309 (36.6)
Substance use disorder		
No	15 562 (83.8)	5981 (38.4)
Yes	3007 (16.2)	456 (15.2)
Schizophrenia spectrum disorder		
No	14 919 (80.3)	4287 (28.7)
Yes	3650 (19.7)	2150 (58.9)
Affective psychotic disorder		
No	17 786 (95.8)	6039 (34.0)
Yes	783 (4.2)	398 (50.8)
Mood disorder		
No	15 737 (84.8)	5812 (36.9)
Yes	2832 (15.3)	625 (22.1)
Stress-related disorder		
No	17 038 (91.8)	6146 (36.1)
Yes	1531 (8.2)	291 (19.0)
Behavioural syndromes		
No	18 313 (98.6)	6390 (34.9)
Yes	256 (1.4)	47 (18.4)
Personality disorder		
No	17 837 (96.1)	6297 (35.3)
Yes	732 (3.9)	140 (19.1)
Mental disability		
No	18 459 (99.4)	6395 (34.6)
Yes	110 (0.6)	42 (38.2)
Psychological development disorder		
No	18 496 (99.6)	6408 (34.7)
Yes	73 (0.4)	29 (39.7)
Disorders with onset in childhood		
No	18 534 (99.8)	6426 (34.7)
Yes	35 (0.2)	11 (31.4)
Service use 12 months prior to admission, *n* (%)		
SLaM appointments (any service)		
0	4502 (24.2)	1402 (31.1)
1–5	6364 (34.3)	2308 (36.3)
6–11	2839 (15.3)	1054 (37.1)
≥12	4864 (26.2)	1673 (34.4)
Home treatment, 12 months prior admission		
No	15 390 (82.9)	5144 (33.4)
Yes	3179 (17.1)	1293 (40.7)
Early intervention for psychosis 12 months prior admission		
No	17 845 (96.1)	6028 (33.8)
Yes	724 (3.9)	409 (56.5)
Psychological therapies, 12 months prior admission		
No	16 923 (91.1)	6017 (35.6)
Yes	1646 (8.9)	420 (25.5)
Care plan 12 months prior admission		
No	14 571 (78.5)	5165 (35.5)
Yes	3998 (21.5)	1272 (31.8)

**IMD, Index of Multiple Deprivation; IQR, interquartile range; MHA, Mental Health Act; SLaM,:** South London and Maudsley NHS Foundation Trust.

**a. For the total column, where:** *n* (%) is presented the % represents the % for the total sample and for the Admitted to hospital under the MHA column the % represents the % for that group.

In the 12 months prior to admission, 24% of the cohort did not have any appointment with SLaM services, 34% had 1–5 appointments, 15% had 6–11 and 26% had ≥12 appointments. In total, 17% received home treatment (at least once), 4% received care from an early intervention for psychosis team, 9% received psychological therapy (i.e. CBT in SLaM or talking therapies via the IAPT services) and 22% had a care plan in place.

### Involuntary admission, ethnicity and service use before admission

About a third (35%) of the first hospital admissions were involuntary. The lowest rates of involuntary admission were observed among White British people (23%), whereas the highest were among people of Asian Chinese (52%), Black African (52%), Black Caribbean (50%) and Black British/Other Black (49%) backgrounds.

Adjusting for demographics and diagnoses, 10 of the 14 minoritised ethnic groups had significantly higher odds of involuntary admission, as opposed to voluntary admission, compared with White British people (Table 2).

**Table 2:** Logistic regression models

	OR (95% CI) for involuntary admission under the MHA		
	Crude	Adjusted for sociodemographics and diagnoses	Fully adjusted^a^
Ethnicity (reference: White British)			
White Irish	1.13 (0.91–1.38)	1.08 (0.86–1.34)	1.09 (0.87–1.36)
Other White	**2.28 (2.05–2.53)**	**1.84 (1.61–2.10)**	**1.84 (1.60–2.10)**
Black African	**3.64 (3.29–4.03)**	**2.38 (2.09–2.71)**	**2.34 (2.05–2.66)**
Black Caribbean	**3.44 (3.03–3.91)**	**2.27 (1.96–2.61)**	**2.27 (1.97–2.60)**
Black British/Other Black	**3.24 (2.92–3.60)**	**2.33 (2.08–2.61)**	**2.32 (2.06–2.60)**
Asian Indian	**1.93 (1.51–2.47)**	**1.56 (1.20–2.04)**	**1.54 (1.18–2.00)**
Asian Pakistani	**2.48 (1.80–3.41)**	**1.83 (1.31–2.58)**	**1.74 (1.23–2.45)**
Asian Bangladeshi	**2.97 (1.96–4.51)**	**2.23 (1.42–3.49)**	**2.23 (1.42–3.50)**
Asian Chinese	**3.73 (2.63–5.28)**	**2.90 (2.00–4.20)**	**2.83 (1.95–4.11)**
Asian British/Other Asian	**2.25 (1.91–2.65)**	**1.78 (1.48–2.14)**	**1.76 (1.46–2.11)**
White and Black African	1.55 (0.97–2.47)	1.08 (0.65–1.79)	1.08 (0.65–1.79)
White and Black Caribbean	**1.45 (1.09–1.94)**	1.31 (0.96–1.78)	1.28 (0.94–1.74)
Other Mixed race	**1.66 (1.20–2.29)**	1.29 (0.92–1.82)	1.29 (0.91–1.82)
Other ethnicity	**2.40 (2.10–2.73)**	**1.85 (1.60–2.16)**	**1.82 (1.57–2.12)**
SLaM out-patient appointments (any service) in the 12 months prior to admission (reference: 0 appointments)			
1–5	**1.25 (1.16–1.36)**	**1.29 (1.18–1.41)**	**1.28 (1.17–1.40)**
6–11	**1.31 (1.18–1.44)**	**1.35 (1.21–1.51)**	**1.34 (1.19–1.50)**
≥12	**1.16 (1.06–1.26)**	**1.18 (1.07–1.30)**	**1.19 (1.06–1.34)**
Home treatment, 12 months prior admission (yes versus no)	**1.37 (1.26–1.48)**	**1.24 (1.14–1.35)**	**1.30 (1.18–1.43)**
Early intervention for psychosis, 12 months prior admission (yes versus no)^b^	**1.33 (1.06–1.66)**	1.15 (0.91–1.45)	**1.34 (1.04–1.73)**
Psychological therapies, 12 months prior admission (yes versus no)	**0.62 (0.55–0.70)**	**0.69 (0.60–0.78)**	**0.66 (0.58–0.75)**
Care plan, 12 months prior admission (yes versus no)	**0.85 (0.79–0.92)**	**0.87 (0.80–0.95)**	**0.77 (0.70–0.85)**

MHA, Mental Health Act; SLaM, South London and Maudsley NHS Foundation Trust.

a. Models adjusted for sociodemographic information and psychiatric diagnoses and all measures of clinical care (number of SLaM appointments, home treatment, early intervention for psychosis, psychological therapies, care plan in place).

b. Regression models are restricted to people with a diagnosis of a schizophrenia spectrum disorder or affective psychosis and people aged up to 65 years (*n* = 3956).

The magnitudes of the observed ethnic inequalities were relatively similar in the model adjusting only for sociodemographic factors and psychiatric dianoses and in the model adjusting for prior clinical care (fully adjusted model). There were observed higher odds for involuntary admission (by decreasing magnitude) among the following minority ethnic groups: Asian Chinese (adjusted odds ratio (aOR) = 2.82, 95% CI 1.94–4.09), Black African (aOR = 2.32, 95% CI 2.04–2.64); Black British (aOR = 2.29 95% CI 2.04–2.57); Black Caribbean (aOR = 2.24, 95% CI 1.95–2.58); Asian Bangladeshi (aOR = 2.23, 95% CI 1.42–3.49); Other White background (aOR = 1.82, 95% CI 1.59–2.08); Other ethnic background (aOR = 1.82, 95% CI 1.56–2.11); Asian British (aOR = 1.75, 95% CI 1.45–2.10); Asian Pakistani (aOR = 1.75, 95% CI 1.24–2.46); and Asian Indian (aOR = 1.54, 95% CI 1.18–2.01).

Significant inequalities in involuntary admission were not observed among people of White Irish and Mixed race ethnicity (any Mixed race background). Sensitivity analyses, restricted to hospital admissions after 2010 (because of potential patchiness in data prior 2010), or including only patients who had an address in the SLaM catchment area at the time of admission, revealed similar findings (Supplementary Table 2).

When adjusting for sociodemographic information and diagnoses, the type and frequency of clinical care were also related to rates of involuntary admission (Table 2). Surprisingly, having had any number of appointments in SLaM was associated with increased odds (of small magnitude) of having an involuntary admission when compared with not having any SLaM appointment (for example for those with 6 to 11 appointments, aOR_6–11_ = 1.34, 95% CI 1.19–1.50).

In the sensitivity analyses, when restricting the cohort to people with a SLaM catchment address at the time of admission (thus, potentially excluding people who were homeless, or receiving care in SLaM regularly but who were living in another London borough) significant differences were observed only for those with 6–11 appointments, as compared to 0 appointments. However, the direction of the effects for any number of appointments was similar to the ones in the main analysis.

Having received support from a home treatment team was also associated with higher odds of involuntary admission (aOR = 1.30, 95% CI 1.18–1.43), in both the main cohort and sensitivity analyses. Receiving care from early intervention or psychosis services was related to higher odds of involuntary admission (aOR = 1.34, 95% CI 1.04–1.73) in the main analysis. When restricting the cohort to a first admission after 2010, the association was no longer significant. This suggests that bias because of potential data patchiness, or inconsistent service provision, before 2010 may be inflating the association. However, the direction of the effects in the sensitivity analyses was similar.

People who received psychological therapies (i.e. CBT or talking therapies in the community (IAPT service)) had 34% reduced odds of involuntary admission (aOR = 0.66, 95% CI 0.58–0.75). Similar associations were observed in the sensitivity analyses (Supplementary Table 2).

Also, a record of a care plan was associated with a 23% reduction in the odds of involuntary admission (aOR = 0.77, 95% CI 0.70–0.85). Similar associations were observed in the sensitivity analyses (Supplementary Table 2).

### Ethnic inequalities in clinical care and mediation effects

**Fig. 2:**
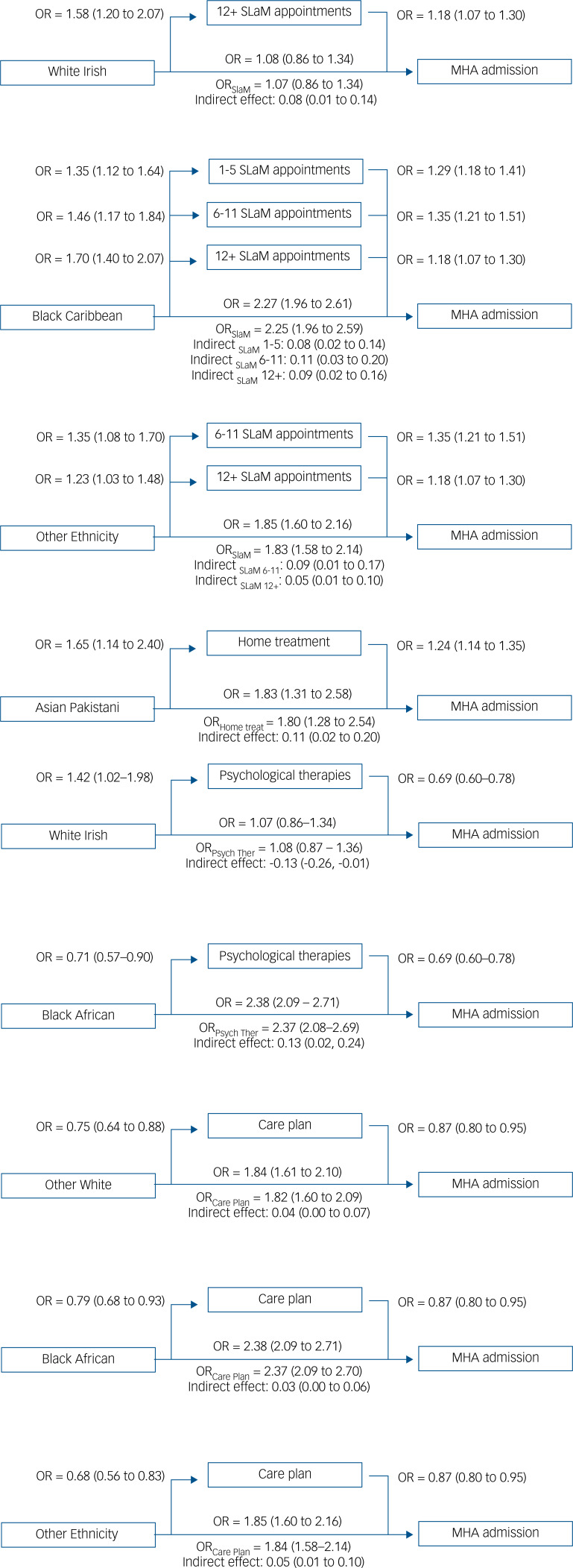
Graphical representation of the path analyses with significant indirect effects between ethnicity and involuntary admission via clinical care. Home treat, home treatment; MHA, Mental Health Act; Psych Ther, psychological therapies; SLaM, South London and Maudsley NHS Foundation Trust.

We observed some ethnic inequalities in clinical care prior to admission, with a significant portion of the effects of ethnicity being mediated via clinical care. Fig. 2 provides a graphical representation of the significant mediation effects observed; complete information on the results is provided in Supplementary Tables 3–7.

In the 12 months before admission, compared with White British people, White Irish people were more likely to have ≥12 appointments; Black Caribbean people had higher odds of having any number of SLaM appointments (1–5, 6–11, ≥12 rather than 0), and people of Other ethnic background were also more likely to have 1–5 and 6–11 appointments (Supplementary Table 3). The significant indirect effect suggests higher odds of involuntary admission with an increased number of appointments. However, there was no substantial change in the magnitude of inequalities in involuntary admission, after adjusting for the number of prior appointments (Fig. 2).

Asian Pakistani people received more home treatment than White British people, which was significantly associated with increased rates of involuntary admission (Supplementary Table 4). But accounting for the effect of home treatment did not substantially change the magnitude of the inequalities. No significant ethnic inequalities in access to early intervention for psychosis services were observed, so no indirect effects were tested (Supplementary Table 5).

Black African people were less likely to have received psychological therapies than White British people, and the significant indirect effect suggests an increase in odds of involuntary admission because of that (Supplementary Table 6). In contrast, White Irish people were more likely to have received psychological therapies, which contributed to reduced involuntary admission. But, accounting for the effect of psychological therapies did not substantially change the magnitude of inequalities.

People with Other White, Black African and Other ethnic backgrounds were less likely to have a care plan. The significant indirect effects suggest increased risk for involuntary admission among these ethnic groups because of the absence of a care plan (Supplementary Table 7). However, the magnitude of inequalities in involuntary admission rates was not substantially altered after adjusting for a care plan.

## Discussion

### Main findings

We investigated ethnic inequalities in involuntary admission under the MHA and prior clinical care received in a cohort of people with a first hospital admission, in a 13-year observation window. Results show that 10 of the 14 minoritised ethnic groups had higher rates of involuntary admission than White British people, with the exceptions being White Irish people and people in the any Mixed race background group. Additionally, we observed that a higher number of SLaM appointments in the 12 months prior to admission (as compared with zero appointments) was related to higher rates of involuntary admission. Home treatment was also related to higher rates of involuntary admission. Care from an early intervention for psychosis team was not consistently related to involuntary admission. Having received psychological therapies and having a care plan was associated with reduced odds of compulsory admission.

There is evidence of some ethnic inequalities in clinical care and the impact of ethnicity being mediated by clinical care. However, although statistically significant indirect effects were observed, the magnitude of the ethnic disparities in involuntary admission was not substantially explained by differences in clinical care prior to admission.

### Ethnicity and involuntary admission

The magnitude of the observed ethnic inequalities in involuntary admission is similar to what has been reported in recent meta-analyses.^8^ Differences observed, compared with previous literature, relate only to the Other White and Mixed race ethnic groups. One study observed that controlling for diagnosis, age and clinical team, men of Other White backgrounds did not have higher odds of involuntary admission than White British men.^20^ However, that study included only people within the first year of receiving care from early intervention for psychosis services, and the comparision group included people who were not hospitalised. Other studies have reported that people of Mixed race ethnicity have almost twice the odds of involuntary admission,^21^ and Mixed race women attending earlier intervention services for psychosis also had higher involuntary admission rates.^20,21^ Again, one major difference between these studies and ours is that their comparison groups include people who were not hospitalised, while our cohort is restricted to people in their first hospital admission episode.

The pattern of ethnic inequalities in involuntary admissions, with only 4 of the 14 minority ethnic groups not having a higher risk than White British, and those groups being White Irish, White and Black African, White and Black Caribbean and Other Mixed race, suggests that social and cultural differences could be affecting the experience and outcomes of care.^22^ There is evidence that people who face multiple forms of disadvantage (such as poverty, poor literacy, unemployment, poor housing and discrimination) may be in a less favourable position than White British people to negotiate their care.^23^ Previous experiences of discrimination and associated greater mistrust in mental health services may also affect the quality of therapeutic relationships.^2,23,24^

### Prior clinical care and involuntary admission

Our results suggest that ethnic disparities in involuntary admission are not because of lack of engagement with services. We observed that people with 6 to 11 appointments, as compared with those with no appointments, had higher odds of involuntary admission (although the magnitude of the effects was small). These findings are against our hypotheses and differ from previous evidence in the literature.^4^ The associations may reflect that illness severity or ongoing clinical crises are undermining the efficacy of care in preventing involuntary admission. A possible explanation may be related to the diversity of teams providing care in the community, with potentially less continuity of care and a weaker therapeutic alliance. A previous study showed that lack of continuity of care is detrimental to clinical outcomes.^25^ Another likely explanation is that the provision of appointments is dependent on perceptions of risk – an unmeasured factor that is highly related to involuntary admission.^4,26–28^ Thus, patients perceived to be at higher risk are provided with more appointments, albeit that an increased number of appointments is not contributing to reduced odds of involuntary admission.

Home treatment is associated with increased risk of involuntary admission. This finding is unexpected, as home treatment is a way to reduce admissions^1^ and a previous review of randomised controlled trials suggests that receiving care from a crisis resolution team is associated with reduced involuntary admissions.^13^ However, there is also evidence of poor experience of home treatment, with some people reporting negative experiences with the teams and the need for more funding for these teams.^1^

Psychological therapies were associated with reduced rates of involuntary admission. A recent review of the use of CBT to prevent involuntary hospital admission revealed no significant protective effects on preventing compulsory admission.^13^ However, the studies included in the review were randomised controlled trials with people with psychosis only. It may be that patients with more complex needs, or acute symptomatic presentations, who are the ones more likely to be involuntary admitted are not getting access to psychological therapies or are refusing them. Concomitantly, a potentially positive effect of psychological therapies can be associated with greater opportunity to develop self-management, a strategy associated with reduced risk of involuntary admission.^13^ It may also be that CBT and talking therapies enhance patients’ trust in the care system and improve therapeutic alliance, leading to voluntary seeking of in-patient care when there is clinical deterioration. Furthermore, our study shows that Black African people were less likely to receive psychological therapies. This is in line with findings from a nationally representative study with people with schizophrenia,^11^ and with evidence that, among people with common mental disorders, people from minoritised ethnic groups were less likely to receive treatment, including counselling.^29^ Another study reports that, after referral for IAPT, several minority ethnic groups were less likely to be assessed.^12^ Among those who did not receive an assessment, Black African people had the highest percentages for declining such assessment.^12^

Having a care plan was protective against involuntary admission. The effect may be related to a patient having the opportunity to discuss the management of their health with their clinicians and thus feel more in control of their clinical care. To our knowledge, this is the first study to show a relationship between having a care plan and involuntary admission. Care plans assessed in this study were not crisis plans, or advanced statements, but there is evidence that formulating an advanced statement is protective of involuntary admission.^15,30^ It may be that some of the underlying mechanisms that confer the protection provided by crisis plans are present during the formulation of common care plans, namely shared decision-making and a good therapeutic alliance. We observed that Other White, Black African and Asian Chinese people were less likely to have a care plan formulated. These findings are partially in line with a previous study with a nationally representative sample of people with schizophrenia; it was observed that Asian/Asian British (excluding Chinese) had reduced odds of having a care plan.^11^

### Limitations and strengths

One limitation regards the non-inclusion of other predictors of involuntary admission, namely social support, marital status and household, and the source of referral to secondary care.^7,9^ About 1% of people that met the inclusion criteria were not included in the cohort as they were part of the national opt-out, and data for about 8% of patients using IAPT services are not linked to CRIS, because of non-disclosure of their NHS number. This may have led to an underrepresentation of people benefiting from psychological therapies.

Another important limitation is the crude assessment of the severity of illness based only on diagnoses. The level of severity of illness could preclude receiving some types of care (such as home treatment, psychological therapies). Thus, we are not able to rule out potential confounding by indication when assessing clinical care prior to admission. Having access to more intensive care (such as a higher number of appointments or home treatment) could be the result of greater severity of illness. Thus, it is likely that people who are given this type of care are already at higher risk for involuntary admission.

Our study strengths include using real-world data to investigate types of care and their relationship with involuntary admission. The study sample is representative of the SLaM's catchment population because of free access to NHS medical care in the UK. Also, the SLaM catchment population is very diverse (the majority of its residents have a minority ethnic background), which allowed investigation of ethnic inequalities without the need to merge distinct ethnic groups into larger categories. Our findings are likely to be generalisable to other larger urban areas in the UK.

### Implications

This study shows that the majority of minoritised ethnic groups (10 of 14) have higher rates of involuntary admission, and these higher rates are not explained by differences in clinical care prior to admission. Future research is needed to uncover the reasons for the observed higher odds of involuntary admission rates among those who have had more SLaM out-patient appointments in the 12 months prior to admission and those who received home treatment. Access to psychological therapies and the formulation of care plans could be promoted as a way to potentially reduce involuntary admission.

## Data Availability

The data from the clinical electronic records is owned by the South London and Maudsley NHS Trust and can be accessed via the Clinical Records Interactive Search. The data are not publicly available due to the Information Governance framework and Research Ethics Committee approval in place concerning CRIS data use. The data that support the findings of this study are available on request from the corresponding author.

## Supplementary material

For supplementary material accompanying this paper visit https://doi.org/10.1192/bjp.2022.141. click here to view supplementary material
