# Supplementary material for: Ethnic inequalities in involuntary admission under the Mental Health Act: an exploration of mediation effects of clinical care prior to the first admission
Source: Br J Psychiatry. 2023 Jan;222(1):27–36. doi: 10.1192/bjp.2022.141 (PMC10250681; doi:10.1192/bjp.2022.141)
Supplement: Supplementary file 1 [file S0007125022001416sup001.docx]

**Supplementary material**

**Study:** Ethnic inequalities in involuntary admission under the Mental Health Act: an exploration of mediation effects of clinical care prior to the first admission

**Authors**: Daniela Fonseca de Freitas, Susan Walker, Patrick Nyikavaranda, Johnny Downs, Rashmi Patel, Mizanur Khondoker, Kamaldeep Bhui & Richard Hayes

**Table S1.** Demographic and clinical characteristics of the cohort of people hospitalised stratified by ethnicity

|  | White British  *n* (%) | White Irish  *n* (%) | Other White  *n* (%) | Black African  *n* (%) | Black Caribbean  *n* (%) | Black British  *n* (%) | Asian Indian  *n* (%) | Asian Pakistani  *n* (%) | Asian Bangladeshi  *n* (%) | Asian Chinese  *n* (%) | Asian British  *n* (%) | White & Black African  *n* (%) | White & Black Caribbean  *n* (%) | Other Mixed Race  *n* (%) | Other Ethnicity  *n* (%) |
| --- | --- | --- | --- | --- | --- | --- | --- | --- | --- | --- | --- | --- | --- | --- | --- |
| Gender: Women | 3,622 (44%) | 203 (39.6) | 800 (43.2) | 977 (43.2) | 529 (46.2) | 785 (41.6) | 131 (45.6) | 85 (53.5) | 42 (46.7) | 94 (72.3) | 262 (39.6) | 43 (51.8) | 116 (51.1) | 83 (48.5) | 454 (41.5) |
| Age |  |  |  |  |  |  |  |  |  |  |  |  |  |  |  |
| 18-24 | 1,018 (12.4) | 32 (6.2) | 285 (16.4) | 395 (19.5) | 125 (10.9) | 539 (28.6) | 32 (11.2) | 42 (26.4) | 15 (16.7) | 35 (26.9) | 126 (19.1) | 24 (28.9) | 61 (26.9) | 49 (28.7) | 228 (20.9) |
| 25-34 | 1,677 (20.4) | 76 (14.8) | 604 (32.6) | 651 (32.1) | 187 (16.3) | 513 (27.2) | 58 (20.2) | 43 (27.0) | 39 (43.3) | 48 (36.9) | 186 (28.1) | 21 (25.3) | 66 (29.1) | 62 (36.3) | 340 (31.1) |
| 35-49 | 2,579 (31.3) | 164 (32.0) | 625 (33.8) | 674 (33.2) | 324 (28.3) | 580 (30.7) | 94 (32.8) | 47 (29.6) | 25 (27.8) | 27 (20.8) | 222 (33.6) | 27 (32.5) | 67 (29.5) | 45 (26.3) | 335 (30.7) |
| 60-64 | 1,591 (19.3) | 98 (19.1) | 203 (11.0) | 229 (11.3) | 255 (22.3) | 221 (11.7) | 57 (19.9) | 16 (10.1) | < 10% | < 10% | 85 (12.9) | < 10% | 21 (9.3) | < 10% | 145 (13.3) |
| 65-99 | 1,375 (16.7) | 143 (27.9) | 135 (7.3) | 82 (4.0) | 254 (22.2) | 34 (1.8) | 46 (16.0) | 11 (6.9) | < 5% | < 10% | 42 (6.4) | < 5% | 12 (5.3) | < 5% | 45 (4.1) |
| Migrant |  |  |  |  |  |  |  |  |  |  |  |  |  |  |  |
| No | 5,476 (66.5) | 103 (20.1) | 113 (6.1) | 131 (6.5) | 234 (20.4) | 934 (49.5) | 69 (24.1) | 36 (22.6) | 19 (21.1) | < 10% | 85 (12.9) | 32 (38.6) | 125 (55.1) | 68 (39.8) | 79 (7.2) |
| Yes | 149 (1.8) | 287 (6.0) | 1,372 (74.1) | 1,348 (66.4) | 369 (32.2) | 231 (12.2) | 119 (41.5) | 77 (48.4) | 46 (51.1) | 94 (72.3) | 391 (59.2) | 28 (33.7) | 18 (7.9) | 37 (21.6) | 533 (48.8) |
| Undetermined | 2,615 (31.7) | 123 (24.0) | 367 (19.8) | 552 (27.2) | 542 (47.3) | 722 (38.3) | 99 (34.5) | 46 (28.9) | 25 (27.8) | < 25 | 185 (28.0) | 23 (27.7) | 84 (37.0) | 66 (38.6) | 481 (44.0) |
| Homeless 12 months prior admission | 1,898 (23.0) | 151 (29.4) | 424 (22.9) | 252 (12.4) | 113 (9.9) | 261 (13.8) | 48 (16.7) | 14 (8.8) | 15 (16.7) | 14 (10.8) | 88 (13.3) | 14 (16.9) | 46 (20.2) | 31 (18.1) | 142 (13.0) |
| Neighbourhood deprivation |  |  |  |  |  |  |  |  |  |  |  |  |  |  |  |
| Least deprived quintile | 1,487 (18.1) | 46 (9.0) | 183 (9.9) | 132 (6.5) | 87 (7.6) | 150 (8.0) | 63 (22.0) | 31 (19.5) | < 10% | 19 (14.6) | 83 (12.6) | < 5% | 20 (8.8) | 25 (14.6) | 102 (9.3) |
| 2^nd^ least deprived | 1,582 (19.2) | 86 (16.8) | 316 (17.1) | 315 (15.5) | 208 (18.2) | 317 (16.8) | 54 (18.8) | 41 (25.8) | 15 (16.7) | 24 (18.5) | 130 (19.7) | < 10% | 48 (21.2) | 33 (19.3) | 191 (17.5) |
| Middle deprivation | 1,485 (18.0) | 89 (17.4) | 372 (20.1) | 413 (20.3) | 238 (20.8) | 386 (20.5) | 53 (18.5) | 25 (15.7) | 11 (12.2) | 22 (16.9) | 122 (18.5) | 22 (26.5) | 51 (22.5) | 37 (21.6) | 240 (22.0) |
| 2^nd^ most deprived | 1,499 (18.2) | 108 (21.1) | 321 (17.3) | 458 (22.6) | 248 (21.7) | 423 (22.4) | 38 (13.2) | 21 (13.2) | 21 (23.3) | 26 (20.0) | 139 (21.0) | 21 (25.3) | 48 (21.2) | 29 (17.0) | 207 (18.9) |
| Most deprived quintile | 1,362 (16.5) | 105 (20.5) | 254 (13.7) | 465 (22.9) | 269 (23.5) | 414 (21.9) | 48 (16.7) | 30 (18.9) | 30 (33.3) | 20 (16.4) | 115 (17.4) | 18 (21.7) | 39 (17.2) | 28 (16.4) | 190 (17.4) |
| Undetermined | 825 (10.0) | 79 (15.4) | 406 (21.9) | 248 (12.2) | 95 (8.3) | 197 (10.4) | 31 (10.8) | 11 (6.9) | < 10% | 19 (14.6) | 72 (10.9) | 12 (14.5) | 21 (9.3) | 19 (11.1) | 163 (14.9) |
| Diagnoses of psychiatric disorders |  |  |  |  |  |  |  |  |  |  |  |  |  |  |  |
| Organic disorder | 475 (5.8) | 47 (9.2) | 57 (3.1) | 98 (8.6) | 43 (2.3) | < 5% | 11 (6.9) | < 5% | < 5% | 12 (1.8) | < 5% | < 5% | < 5% | < 5% | 29 (2.7) |
| Substance use disorder | 1,806 (21.9) | 137 (26.7) | 282 (15.2) | 148 (7.3) | 86 (7.5) | 234 (12.4) | 50 (17.4) | < 10% | < 15% | < 10% | 47 (7.1) | 12 (14.4) | 47 (20.7) | 21 (12.3) | 110 (10.1) |
| Schizophrenia spectrum disorder | 886 (10.8) | 49 (9.6) | 461 (25.9) | 679 (33.4) | 301 (26.3) | 610 (32.3) | 55 (19.2) | 39 (24.5) | 28 (31.1) | 32 (24.6) | 148 (22.4) | 26 (21.3) | 36 (15.9) | 38 (22.2) | 262 (24.0) |
| Affective psychotic disorder | 290 (3.5) | 18 (3.5) | 95 (5.1) | 100 (4.9) | 47 (4.1) | 74 (3.9) | 19 (6.6) | 20 (12.6) | < 10% | < 5% | 33 (5.0) | < 10% | < 5% | < 5% | 59 (5.4) |
| Mood disorder | 1,459 (17.7) | 81 (15.8) | 298 (16.1) | 196 (9.7) | 123 (10.7) | 191 (10.1) | 46 (16.0) | 26 (16.4) | 17 (18.9) | 32 (24.6) | 110 (16.6) | 13 (16.7) | 35 (15.4) | 14 (8.2) | 191 (17.5) |
| Stress related | 796 (9.7) | 28 (5.5) | 130 (7.0) | 125 (6.2) | 46 (4.0) | 110 (5.8) | 15 (5.2) | 20 (12.6) | < 5% | < 10% | 97 (14.7) | < 10% | 14 (6.2) | 12 (7.0) | 120 (11.0) |
| Behavioural syndromes | 152 (1.8) | < 5% | 26 (1.4) | 18 (0.9) | < 5% | < 5% | < 5% | < 5% | < 5% | < 5% | < 5% | < 5% | < 5% | < 5% | < 5% |
| Personality disorder | 425 (5.2) | 16 (3.1) | 55 (3.0) | 22 (1.1) | 17 (1.5) | 63 (3.3) | < 5% | < 5% | < 5% | < 5% | 21 (3.2) | < 5% | 16 (7.1) | 15 (8.8) | 65 (6.0) |
| Mental disability | 52 (0.6) | < 5% | < 5% | 11 (0.6) | < 5% | 18 (1.0) | < 5% | < 5% | < 5% | < 5% | < 5% | < 5% | < 5% | < 5% | < 5% |
| Psychological developmental disorder | 39 (0.5) | < 5% | < 5% | < 5% | < 5% | 12 (0.6) | < 5% | < 5% | < 5% | < 5% | < 5% | < 5% | < 5% | < 5% | < 5% |
| Disorders with onset in childhood | 18 (0.2) | < 5% | < 5% | < 5% | < 5% | < 5% | < 5% | < 5% | < 5% | < 5% | < 5% | < 5% | < 5% | < 5% | < 5% |
| SLaM appointments (any service) |  |  |  |  |  |  |  |  |  |  |  |  |  |  |  |
| 0 | 1,866 (22.7) | 101 (19.7) | 555 (30.0) | 601 (29.6) | 195 (17.0) | 468 (24.8) | 57 (19.9) | 37 (23.3) | 27 (30.0) | 43 (33.1) | 177 (26.8) | 21 (25.3) | 49 (21.6) | 35 (20.5) | 270 (24.7) |
| 1-5 | 2,742 (33.3) | 149 (29.0) | 679 (36.7) | 705 (34.7) | 372 (32.5) | 644 (34.1) | 92 (32.1) | 64 (40.3) | 34 (37.8) | 39 (30.0) | 258 (39.0) | 26 (31.3) | 64 (28.2) | 67 (39.2) | 429 (39.3) |
| 6-11 | 1,287 (15.6) | 83 (16.2) | 253 (13.7) | 290 (14.3) | 182 (16.2) | 288 (15.3) | 52 (18.1) | 30 (18.9) | 11 (12.2) | 20 (15.4) | 88 (13.3) | 13 (16.7) | 40 (17.6) | 24 (14.0) | 175 (16.0) |
| 12+ | 2,345 (28.5) | 180 (35.1) | 365 (19.7) | 435 (21.4) | 393 (34.3) | 487 (25.8) | 86 (30.0) | 28 (17.6) | 18 (20.0) | 28 (21.5) | 138 (20.9) | 23 (27.7) | 74 (32.6) | 45 (26.3) | 219 (20.0) |
| Home treatment | 1,428 (17.3) | 87 (17.0) | 251 (13.6) | 347 (17.1) | 219 (19.1) | 310 (16.4) | 57 (19.9) | 42 (26.4) | 14 (15.6) | 24 (18.5) | 130 (19.7) | 14 (16.9) | 45 (19.8) | 31 (18.1) | 180 (16.5) |
| Early intervention for psychosis | 164 (2.0) | < 5% | 60 (3.2) | 137 (6.8) | 53 (4.6) | 149 (7.9) | 13 (4.5) | < 5% | < 5% | < 5% | 27 (4.1) | < 10% | 19 (8.4) | < 5% | 62 (5.7) |
| Psychological therapies | 795 (9.7) | 45 (8.8) | 134 (7.2) | 118 (5.8) | 80 (7.0) | 191 (10.1) | 23 (8.0) | 11 (6.9) | < 10% | < 5% | 60 (9.1) | 10 (12.1) | 27 (11.9) | 16 (9.4) | 125 (11.4) |
| Care plan | 2,011 (24.4) | 149 (29.0) | 285 (15.4) | 336 (16.5) | 325 (28.4) | 361 (19.1) | 73 (25.4) | 25 (15.7) | 17 (18.9) | 17 (13.1) | 122 (18.5) | 18 (21.7) | 58 (25.6) | 45 (26.3) | 156 (14.3) |

Note: In cells where the number of people was smaller than 10, we present the closest % in multiples of 5.

**Table S2.** Sensitivity analyses, restricting cohort to people admitted after 2010, and with an address in SLaM catchment at time of admission (*n* = 10,970)

|  | **Admissions after 2010**  **Adjusted OR [95% CI]^a^ (N=14,831)** | **Address in catchment**  **Adjusted OR [95% CI]^a^ (N=13,526)** |
| --- | --- | --- |
| Ethnicity (reference: White British) |  |  |
| White Irish | 1.10 [0.85 – 1.43] | 1.12 [0.86 – 1.45] |
| Other White | **1.74 [1.50 – 2.03]** | **1.77 [1.50 – 2.09]** |
| Black African | **2.30 [2.00 – 2.66]** | **2.33 [2.00 – 2.71]** |
| Black Caribbean | **2.25 [1.92– 2.63]** | **2.24 [1.92 – 2.63]** |
| Black British / Other Black | **2.14 [1.89 – 2.43]** | **2.11 [1.94 – 2.52]** |
| Asian Indian | **1.40 [1.04 – 1.89]** | **1.83 [1.35 – 2.49]** |
| Asian Pakistani | **1.61 [1.12 – 2.31]** | **1.94 [1.32 – 2.87]** |
| Asian Bangladeshi | **2.49 [1.52 – 4.09]** | **2.14 [1.26 – 3.61]** |
| Asian Chinese | **3.11 [2.05 – 4.71]** | **2.34 [1.51 – 3.61]** |
| Asian British / Other Asian | **1.69 [1.38 – 2.08]** | **1.73 [1.40 – 2.14]** |
| White and Black African | 1.17 [0.69 – 1.99] | 1.28 [0.72 – 2.25] |
| White and Black Caribbean | 1.30 [0.93 – 1.82] | 1.10 [0.77 – 1.58] |
| Other Mixed Race | 1.27 [0.88 – 1.83] | 1.50 [1.00 – 2.25] |
| Other Ethnicity | **1.71 [1.45 – 2.01]** | **1.75 [1.46 – 2.09]** |
| SLaM appointments (any service) (reference: 0 appointments) |  |  |
| 1-5 | **1.27 [1.15 – 1.40]** | 1.10 [0.99 – 1.23] |
| 6-11 | **1.40 [1.23 – 1.58]** | **1.17 [1.02 – 1.34]** |
| 12+ | **1.19 [1.04 – 1.35]** | 1.07 [0.94 – 1.23] |
| Home treatment, 12 months prior admission (yes vs no) | **1.23 [1.10 – 1.37]** | **1.28 [1.15 – 1.42]** |
| Early intervention for psychosis 12 months prior admission (yes vs no)^b^ | 1.25 [0.95 – 1.62] | **1.33 [1.02 – 1.76]** |
| Psychological therapies, 12 months prior admission (yes vs no) | **0.64 [0.56 – 0.72]** | **0.63 [0.55 – 0.73]** |
| Care plan 12 months prior admission (yes vs no) | **0.86 [0.77 – 0.97]** | **0.73 [0.66 – 0.82]** |

Notes: ^a^ Models adjusted for sociodemographic information and psychiatric diagnoses.

^b^ Regression models are restricted to people with a diagnosis of a schizophrenia spectrum disorder or affective psychosis and people aged up to 65 years (admission after 2010: *n* = 3,495; address in catchment: *n* = 3,003)

Clinical care indicators

Path a

Path b

Involuntary admission

Path c

Ethnicity

Path c’ (adjusted for clinical care)

Controls (sociodemographic and diagnostic information)

**Figure S1:**

*Path a* describes the association of ethnicity with each indicator of clinical care. *Path b* describes the association of each indicator of clinical care with involuntary admission. *Path c* describes the association of ethnicity with involuntary admission, while *path c’* the association of ethnicity with involuntary admission adjusting for the effect of clinical care. All paths were adjusted for sociodemographic and diagnostic information

**Table S3**. Results from the multinomial regression analyses predicting number of appointments in the 12 months (compared to 0 appointments) before admission and the indirect effect of ethnicity on involuntary admission via number of appointments.

|  | **Ethnicity predicting 1-5 appointments (*path a* in the mediation diagram)**  **RR [95%CI]** | **Indirect effects for having 1-5 appointments** | **Ethnicity predicting 6-11 appointments (*path a* in the mediation diagram)**  **RR [95%CI]** | **Indirect effects for having 6-11 appointments** | **Ethnicity predicting 12+ appointments (*path a* in the mediation diagram)**  **RR [95%CI]** | **Indirect effects**  **for having 12+ appointments** | **1-5 appointments predicting MHA admission**  **(*path b* in the mediation diagram),**  **OR [95%CI]** | **6-11 appointments predicting involuntary**  **(*path b* in the mediation diagram)**  **OR [95%CI]** | **12+ appointments predicting involuntary**  **(*path b* in the mediation diagram)**  **OR [95%CI]** | **Ethnicity predicting MHA admission (*path c* in the mediation diagram)**  **OR [95%CI]** | **Ethnicity predicting MHA admission adjusting for**  **appointments**  **(*path c’* in the mediation diagram)**  **OR [95%CI]** |
| --- | --- | --- | --- | --- | --- | --- | --- | --- | --- | --- | --- |
| White British | Reference |  | Reference |  | Reference |  | **1.29**  **[1.18 – 1.41]** | **1.35**  **[1.21 – 1.51]** | **1.18**  **[1.07 – 1.30]** | Reference | Reference |
| White Irish | 1.02  [0.77 – 1.34] |  | 1.24  [0.91 – 1.71] |  | **1.58**  **[1.20 – 2.07]** | **0.08**  **[0.01, 0.14]** |  |  |  | 1.08  [0.86 – 1.34] | 1.07  [0.86 – 1.34] |
| Other White | 0.93  [0.80 – 1.09] |  | 0.94  [0.77 – 1.15] |  | **0.83**  **[0.70 – 1.00]** | -0.03  [-0.07, 0.01] |  |  |  | **1.84**  **[1.61 – 2.10]** | **1.84**  **[1.61 – 2.11]** |
| Black African | 0.93  [0.80 – 1.09] |  | 1.03  [0.84 – 1.25] |  | 0.87 [0.73 – 1.03] |  |  |  |  |  |  |
| Black Caribbean | **1.35**  **[1.12 – 1.64]** | **0.08**  **[0.02, 0.14]** | **1.46**  **[1.17 – 1.84]** | **0.11**  **[0.03, 0.20]** | **1.70**  **[1.40 – 2.07]** | **0.09**  **[0.02, 0.16]** |  |  |  | **2.27**  **[1.96 – 2.61]** | **2.25**  **[1.96 – 2.59]** |
| Black British / Other Black | 1.04  [0.90 – 1.20] |  | 1.15  [0.97 – 1.37] |  | 1.03  [0.89 – 1.20] |  |  |  |  |  |  |
| Asian Indian | 1.18  [0.84 – 1.66] |  | **1.56**  **[1.05 – 2.31]** | 0.13  [-0.02, 0.29] | **1.48**  **[1.04 – 2.10]** | 0.07  [-0.02, 0.15] |  |  |  | **1.56**  **[1.20 – 2.04]** | **1.54**  **[1.18 – 2.02]** |
| Asian Pakistani | 1.27  [0.84 – 1.93] |  | 1.53  [0.93 – 2.53] |  | 0.85  [0.51 – 1.41] |  |  |  |  |  |  |
| Asian Bangladeshi | 0.97  [0.58 – 1.62] |  | 0.79  [0.39 – 1.62] |  | 0.70  [0.38 – 1.30] |  |  |  |  |  |  |
| Asian Chinese | 0.69  [0.44 – 1.09] |  | 0.99  [0.57 – 1.73] |  | 0.83  [0.50 – 1.38] |  |  |  |  |  |  |
| Asian British / Other Asian | 1.13  [0.91 – 1.40] |  | 1.04  [0.78 – 1.38] |  | 0.95  [0.74 – 1.22] |  |  |  |  |  |  |
| White and Black African | 0.92  [0.51 – 1.65] |  | 1.21  [0.60 – 2.46] |  | 1.18  [0.64 – 2.17] |  |  |  |  |  |  |
| White and Black Caribbean | 0.96  [0.66 – 1.40] |  | 1.43  [0.93 – 2.20] |  | 1.40  [0.96 – 2.05] |  |  |  |  |  |  |
| Other Mixed Race | 1.43  [0.94 – 2.17] |  | 1.28  [0.75 – 2.18] |  | 1.28  [0.81 – 2.05] |  |  |  |  |  |  |
| Other Ethnicity | **1.23**  **[1.03 – 1.48]** | **0.05**  **[0.01, 0.10]** | **1.35**  **[1.08 – 1.70]** | **0.09**  **[0.01, 0.17]** | 1.00  [0.80 – 1.23] | -0.00  [-0.04, 0.04] |  |  |  | **1.85**  **[1.60 – 2.16]** | **1.83**  **[1.58 – 2.14]** |

Note: The OR for ethnicity on admission, adjusting for number of SLaM appointments (mediation path *c’*) and indirect effects were calculated only for the ethnic groups that had a significant association with number of appointments. All analyses were adjusted for all sociodemographic information and diagnoses. Indirect effects were calculated via generalised structural equation modelling of the diagram portrayed on Figure S1. Indirect effects were tested with bootstrap simulation based on 200 replications. Coefficients for indirect effects are unstandardised.

**Table S4.** Results from logistic regression analyses for receiving home treatment in the 12 months before admission and the indirect effect of ethnicity on involuntary admission via home treatment

|  | **Ethnicity predicting Home treatment**  **(*path a* in the mediation diagram)**  **OR [95% CI]** | **Indirect effect of ethnicity on involuntary admission, via home treatment ^a^** | **Home treatment predicting involuntary admission**  **(*path b* in the mediation diagram)**  **OR [95%CI]** | **Ethnicity predicting involuntary admission (*path c* in the mediation diagram)**  **OR [95%CI]** | **Ethnicity predicting involuntary admission adjusting for**  **home treatment**  **(*path c’* in the mediation diagram)**  **OR [95%CI]** |
| --- | --- | --- | --- | --- | --- |
| White British | Reference | Reference | **1.24 [1.14 – 1.35]** | Reference | Reference |
| White Irish | 1.10 [0.85 – 1.42] |  |  |  |  |
| Other White | 0.90 [0.75 – 1.07] |  |  |  |  |
| Black African | 1.08 [0.92 – 1.26] |  |  |  |  |
| Black Caribbean | 1.01 [0.85 – 1.20] |  |  |  |  |
| Black British / Other Black | 0.98 [0.85 – 1.14] |  |  |  |  |
| Asian Indian | 1.24 [0.91 – 1.69] |  |  |  |  |
| Asian Pakistani | **1.65 [1.14 – 2.40]** | **0.11 [0.02, 0.20]** |  | **1.83 [1.31 – 2.58]** | **1.80 [1.28 – 2.54]** |
| Asian Bangladeshi | 0.88 [0.49 – 1.59] |  |  |  |  |
| Asian Chinese | 1.07 [0.67 – 1.70] |  |  |  |  |
| Asian British / Other Asian | 1.24 [0.99 – 1.54] |  |  |  |  |
| White and Black African | 1.02 [0.56 – 1.85] |  |  |  |  |
| White and Black Caribbean | 1.32 [0.93 – 1.85] |  |  |  |  |
| Other Mixed Race | 1.19 [0.79 – 1.78] |  |  |  |  |
| Other Ethnicity | 0.99 [0.82 – 1.20] |  |  |  |  |

Note: All analyses were adjusted for all sociodemographic information and diagnoses. Indirect effects were calculated via generalised structural equation modelling of the diagram portrayed on Figure S1. Indirect effects were tested with bootstrap simulation based on 200 replications. Coefficients for indirect effects are unstandardised.

**Table S5.** Results from logistic regression analyses for receiving care from an Early Intervention for Psychosis team in the 12 months before admission and the indirect effect of ethnicity on involuntary admission via early intervention for psychosis

|  | **Ethnicity predicting Early Intervention for Psychosis**  **OR [95% CI]** | **Early Intervention for Psychosis predicting involuntary admission**  **(*path b* in the mediation diagram)**  **OR [95%CI]** |
| --- | --- | --- |
| White British | Reference | 1.15 [0.91 – 1.45] |
| White Irish | 2.08 [0.73 – 5.91] |  |
| Other White | 0.93 [0.57 – 1.53] |  |
| Black African | 1.17 [0.77 – 1.75] |  |
| Black Caribbean | 1.43 [0.87 – 2.34] |  |
| Black British / Other Black | 1.11 [0.78 – 1.59] |  |
| Asian Indian | 1.62 [0.63 – 4.17] |  |
| Asian Pakistani | 0.79 [0.26 – 2.36] |  |
| Asian Bangladeshi | 0.66 [0.15 – 2.94] |  |
| Asian Chinese | 0.88 [0.24 – 3.15] |  |
| Asian British / Other Asian | 1.05 [0.56 – 1.97] |  |
| White and Black African | 1.58 [0.50 – 5.03] |  |
| White and Black Caribbean | 2.15 [0.87 – 5.35] |  |
| Other Mixed Race | 0.35 [0.08 – 1.51] |  |
| Other Ethnicity | 1.29 [0.80 – 2.11] |  |

Note: Analyses were adjusted for all sociodemographic information and diagnoses and were restricted to people with a diagnosis of a schizophrenia spectrum disorder or affective psychosis and aged up to 65 years (*n* = 3,929). No indirect effects are presented, as no significant associations of ethnicity with Early Intervention for Psychosis teams were observed.

**Table S6.** Results from the logistic regression analyses psychological therapies in the 12 months before admission and the indirect effect of ethnicity on involuntary admission via psychological therapies

|  | **Ethnicity predicting psychological therapies**  **(*path a* in the mediation diagram)**  **OR [95% CI]** | **Indirect effect of ethnicity on involuntary admission, via psychological therapies ^a^** | **Psychological therapies predicting involuntary admission**  **(*path b* in the mediation diagram)**  **OR [95%CI]** | **Ethnicity predicting involuntary admission (*path c* in the mediation diagram)**  **OR [95%CI]** | **Ethnicity predicting involuntary admission adjusting for psychological therapies ^a^**  **(*path c’* in the mediation diagram)**  **OR [95%CI]** |
| --- | --- | --- | --- | --- | --- |
| White British | Reference | Reference | **0.69 [0.60 – 0.78]** | Reference | Reference |
| White Irish | **1.42 [1.02 – 1.98]** | **-0.13 [-0.26, -0.01]** |  | 1.08 [0.86 – 1.34] | 1.08 [0.87 – 1.36] |
| Other White | 0.97 [0.77 – 1.21] |  |  |  |  |
| Black African | **0.71 [0.57 – 0.90]** | **0.13 [0.02, 0.24]** |  | **2.38 [2.09 – 2.71]** | **2.37 [2.08 – 2.69]** |
| Black Caribbean | 0.90 [0.70 – 1.15] |  |  |  |  |
| Black British / Other Black | 1.03 [0.87 – 1.24] |  |  |  |  |
| Asian Indian | 0.97 [0.62 – 1.52] |  |  |  |  |
| Asian Pakistani | 0.65 [0.34 – 1.22] |  |  |  |  |
| Asian Bangladeshi | 0.70 [0.30 – 1.64] |  |  |  |  |
| Asian Chinese | **0.38 [0.15 – 0.93]** | 0.37 [-0.4, 0.78] |  | **2.90 [2.00 – 4.20]** | **2.84 [1.96 – 4.12]** |
| Asian British / Other Asian | 1.00 [0.74 – 1.34] |  |  |  |  |
| White and Black African | 1.33 [0.67 – 2.64] |  |  |  |  |
| White and Black Caribbean | 1.14 [0.75 – 1.73] |  |  |  |  |
| Other Mixed Race | 0.91 [0.54 – 1.56] |  |  |  |  |
| Other Ethnicity | **1.26 [1.00 – 1.58]** | -0.09 [-0.17, -0.00] |  | **1.85 [1.60 – 2.16]** | **1.87 [1.61 – 2.17]** |

Note: All analyses were adjusted for all sociodemographic information and diagnoses. Indirect effects were calculated via generalised structural equation modelling of the diagram portrayed on Figure S1. Indirect effects were tested with bootstrap simulation based on 200 replications. Coefficients for indirect effects are unstandardised.

**Table S7.** Results from logistic regression analyses for having a care plan formulated and the indirect effect of ethnicity on involuntary admission via a care plan in the 12 months before admission

|  | **Ethnicity predicting having a care plan (*path a* in the mediation diagram)**  **OR [95% CI]** | **Indirect effect of ethnicity on involuntary admission, via having a care plan** | **Care plan predicting involuntary admission**  **(*path b* in the mediation diagram)**  **OR [95%CI]** | **Ethnicity predicting involuntary admission (*path c* in the mediation diagram)**  **OR [95%CI]** | **Ethnicity predicting involuntary admission adjusting for**  **having a care plan**  **(*path c’* in the mediation diagram)**  **OR [95% CI]** |
| --- | --- | --- | --- | --- | --- |
| White British | Reference | Reference | **0.87 [0.80 – 0.95]** | Reference | Reference |
| White Irish | **1.26 [1.02 – 1.56]** | -0.03 [-0.07, 0.01] |  | 1.08 [0.86 – 1.34] | 1.08 [0.86 – 1.35] |
| Other White | **0.75 [0.64 – 0.88]** | **0.04 [0.00, 0.07]** |  | **1.84 [1.61 – 2.10]** | **1.82 [1.60 – 2.09]** |
| Black African | **0.79 [0.68 – 0.93]** | **0.03 [0.00, 0.06]** |  | **2.38 [2.09 – 2.71]** | **2.37 [2.09 – 2.70]** |
| Black Caribbean | **1.24 [1.07 – 1.44]** | -0.03 [-0.06, 0.00] |  | **2.27 [1.96 – 2.61]** | **2.28 [2.00 – 2.63]** |
| Black British / Other Black | 0.89 [0.78 – 1.02] |  |  |  |  |
| Asian Indian | 1.14 [0.86 – 1.51] |  |  |  |  |
| Asian Pakistani | 0.72 [0.46 – 1.11] |  |  |  |  |
| Asian Bangladeshi | 0.88 [0.51 – 1.50] |  |  |  |  |
| Asian Chinese | **0.58 [0.34 – 0.99]** | 0.07 [-0.02, 0.17] |  | **2.90 [2.00 – 4.20]** | **2.88 [1.98 – 4.17]** |
| Asian British / Other Asian | 0.91 [0.73 – 1.13] |  |  |  |  |
| White and Black African | 1.06 [0.62 – 1.81] |  |  |  |  |
| White and Black Caribbean | 1.18 [0.86 – 1.60] |  |  |  |  |
| Other Mixed Race | 1.30 [0.91 – 1.85] |  |  |  |  |
| Other Ethnicity | **0.68 [0.56 – 0.83]** | **0.05 [0.01, 0.10]** |  | **1.85 [1.60 – 2.16]** | **1.84 [1.58 – 2.14]** |

Note: All analyses were adjusted for all sociodemographic information and diagnoses. Indirect effects were calculated via generalised structural equation modelling of the diagram portrayed on Figure S1. Indirect effects were tested with bootstrap simulation based on 200 replications. Coefficients for indirect effects are unstandardised.
